# Diurnal variation of flow-mediated dilatation in healthy humans

**DOI:** 10.1186/s40885-015-0019-4

**Published:** 2015-05-28

**Authors:** Yong Cheol Kim, Kyeong Ho Yun, Sun Ho Woo, Young Hoon Jeong, Jae Hong Lim, Kyo Bum Hwang, Jin Woo Jeong, Mi Rim Lee, Jeong Mi Lee, Sang Jae Rhee, Nam-Ho Kim, Seok Kyu Oh, Jin-Won Jeong

**Affiliations:** Departments of Cardiovascular Medicine, Wonkwang University School of Medicine and Hospital, 895 Muwang-ro, Iksan, Jeonbuk 570-711 Korea; Regional Cardiocerebrovascular Center, Wonkwang University School of Medicine and Hospital, 895 Muwang-ro, Iksan, Jeonbuk 570-711 Korea; Department of Public Health, Wonkwang University Graduate School, Iksan, Korea

**Keywords:** Flow-mediated dilatation, Diurnal variation, Circadian variation

## Abstract

**Introduction:**

The measurement of flow-mediated dilatation (FMD) via ultrasound has been established as a reliable non-invasive measurement of endothelial function. However, the guidelines mention nothing regarding diurnal variation of FMD. Thus, we investigated the FMD in healthy people and diurnal variation of FMD.

**Methods:**

Twenty-five apparently healthy persons participated in this study. All participants had no history of cardiovascular diseases, hypertension, or diabetes and used any medication. For each volunteer, the measurements were repeated in the morning and afternoon on two different days. We checked capillary blood glucose, total cholesterol, triglyceride, high-density lipoprotein (HDL), and low-density lipoprotein (LDL)-cholesterol.

**Results:**

The average of FMD measurements was 8.45% ± 2.39%. The mean values of systolic and diastolic blood pressure, heart rate, lipid profiles, and glucose levels were similar between the morning and afternoon measurements after 9-h fasting. There was no significant difference of FMD measurements between the morning and afternoon (8.32% ± 2.27% and 8.58% ± 2.56%, *p* = 0.329). Moreover, there was significant correlation between FMD in the morning and afternoon (*r* = 0.856, *p* < 0.001).

**Conclusions:**

Our study shows measurement of FMD was 8.45% in healthy Koreans. Also, there was no significant difference of FMD measurements between the morning and afternoon.

## Introduction

Endothelial dysfunction is a systemic disorder and an important factor in the pathogenesis of atherosclerosis, hypertension, and heart failure [1-3]. Impaired endothelial function is associated with increased risk for cardiovascular events [4-6]. The measurement of flow-mediated dilatation (FMD) via ultrasound has been established as a reliable non-invasive measurement of endothelial function [7]. As FMD becomes more and more important in clinical practice to evaluate the risk of coronary heart disease, reproducibility should be available, preferably during the course of the whole day for practical reasons. Reported by the guidelines for the ultrasound assessment endothelial-dependent flow-mediated vasodilation of the brachial artery to decrease sensitivity and establish more reliable FMD measurements, smoking, vitamin supplementation, alcohol intake, and food intake should be controlled [8-12]. However, the guidelines mention nothing regarding a diurnal variation of FMD. Since only a few studies showed that endothelial-dependent FMD had diurnal variation, it was unclear whether there was the effect of the time of measurement on FMD outcome or not [13-15]. Furthermore, it is lack of data about FMD and the absolute values obtained vary considerably across studies in Korea [16-18].

Thus, the purposes of this study were (1) to examine the FMD in Korean and (2) to investigate diurnal variation of FMD.

## Methods

### Study population

Twenty-five apparently healthy persons participated in this study. We evaluated physical history and clinical history including age, gender, weight, height, body mass index (BMI), blood pressure, heart rate, and history of smoking. All participants had no history of cardiovascular diseases, hypertension, or diabetes and used any medication or vitamin preparation. The Institutional Review Board of Wonkwang University Hospital approved the study, and all participants agreed to participate after reading a detailed informed consent form (WKUH 201412-HRE-093).

### FMD measurements

We measured FMD of brachial artery according to the International Brachial Artery Reactivity Task Force guidelines using an ultrasound system equipped with an edge-tracking system for two-dimensional (2D) image and a pulsed Doppler flow velocimeter for automatic measurement (UNEXEF; Unex Co. Ltd., Nagoya, Japan) [8]. The system is comprised of a 10-MHz linear array transducer probe. To control for confounding variables, prior to testing, subjects were instructed to fast and abstain from exercise, caffeine, and tobacco for at least 6 h. For each volunteer, the measurements were repeated at 08:00 to 10:00 and 16:00 to 18:00 on two different days. One observer measured FMD of brachial artery in our study. The whole study was performed by subjects rested in the supine position for 10 min before each measurement. Vascular measurements were performed in the supine position in a quiet, temperature-controlled room of the echocardiography laboratory. Initially, longitudinal 2D images were acquired. After the baseline recording of brachial artery diameter, the blood pressure cuff placed on the subject’s right forearm was inflated 50 mmHg above their systolic blood pressure, leading to arterial occlusion. The diameter of the artery was monitored continuously at the same point, and the maximum dilatation from 90 s after deflation was recorded. FMD was calculated as the difference between the maximum post-occlusive diameter and the baseline diameter, relative to the baseline diameter and expressed as a percentage.

### Laboratory measurement

Blood samples were drawn in the morning and afternoon after at least 6 h fasting, respectively. Before measuring FMD, blood sampling was done. On both occasions, we checked capillary blood glucose, total cholesterol, triglyceride, high-density lipoprotein (HDL), and low-density lipoprotein (LDL) by using test kit (Lipid Pro; Infopia Co. Ltd., Korea).

### Statistical analysis

All measurements were expressed as mean ± standard deviation (SD) or absolute number (percentage). Student *t*-test was used to test for significant differences where appropriate. Pearson’s correlation was used to test bivariate correlations as well. A *P* value <0.05 was considered as significant. Statistical analyses were performed using SPSS 19.0 for windows (SPSS Inc., Chicago IL, USA).

## Results

### Subject characteristics

The study population consists of thirteen male and twelve female subjects. The mean age of the subjects was 35.1 ± 9.8 years. They had a mean BMI of 23.4 ± 2.3 kg/m^2^. There were nine current smokers. The mean values of systolic and diastolic blood pressure, heart rate, lipid profiles, and glucose levels were similar between the morning and afternoon measurements after 9 h fasting (Table 1). Only fasting period was longer in the morning group than afternoon (10.9 ± 1.9 vs 9.2 ± 2.6 h, *p* = 0.008).

**Table 1:** **Subject characteristics (** ***n*** **= 25)**

	**Morning measurement**	**Afternoon measurement**	***p*** **value**
Systolic blood pressure (mmHg)	115.9 ± 9.6	115.5 ± 10.8	0.901
Diastolic blood pressure (mmHg)	73.6 ± 6.4	73.2 ± 7.3	0.837
Heart rate (beat/min)	70.7 ± 9.0	65.6 ± 9.3	0.580
Total cholesterol (mg/dL)	159.1 ± 34.9	170.0 ± 32.2	0.284
Triglyceride (mg/dL)	162.8 ± 72.0	135.7 ± 56.0	0.207
HDL-cholesterol (mg/dL)	39.2 ± 12.8	42.6 ± 15.8	0.465
LDL-cholesterol (mg/dL)	93.5 ± 32.5	107.4 ± 34.4	0.297
Glucose (mg/dL)	103.7 ± 9.7	105.1 ± 6.8	0.547
Fasting period (h)	10.9 ± 1.9	9.2 ± 2.6	0.008

HDL, high density lipoprotein; LDL, low density lipoprotein.

### Brachial artery characteristics

The brachial artery characteristics in the morning and afternoon measurements are shown Table 2. The average brachial artery diameter and FMD measurements were 3.61 ± 0.42 mm and 8.45% ± 2.39%. FMD measurements in the morning and afternoon were 8.32% ± 2.27% and 8.58% ± 2.56%, respectively. There was no significant difference of FMD measurements between the morning and afternoon. The mean ± SD difference between FMD in the morning and afternoon was – 0.26% ± 1.32%. There was also no significant difference between the brachial artery diameter in the two recordings (− 0.01 ± 0.09 mm).

**Table 2:** **Brachial artery characteristics**

	**Morning measurement**	**Afternoon measurement**	***p*** **value**
Brachial artery diameter (mm)	3.61 ± 0.41	3.61 ± 0.45	0.946
Peak artery diameter (mm)	3.90 ± 0.40	3.91 ± 0.44	0.981
Flow-mediated dilatation (FMD) (%)	8.32 ± 2.27	8.58 ± 2.56	0.329

**Figure 1:**
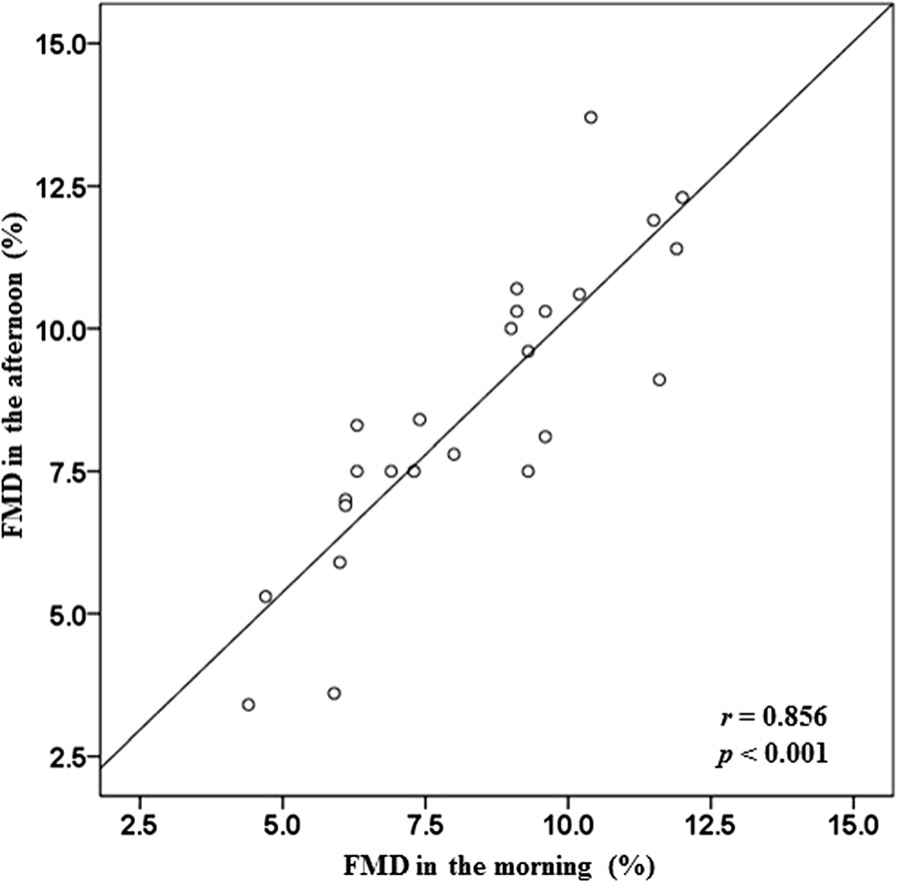
Correlation between flow-mediated dilatation (FMD) in the morning and afternoon. *r*, correlation coefficient, *p*, *p* value by Pearson’s correlation analysis.

In the correlation analysis, there was significant correlation between FMD in the morning and afternoon (*r* = 0.856, *p* < 0.001) (Figure 1). Similarly, brachial artery diameter revealed a significant correlation between both recordings (*r* = 0.983, *p* < 0.001).

## Discussion

In this study, our data shows that the average of FMD was about 8.5%. Moreover, there was no significant difference and significant correlation between FMD in the morning and afternoon. In Korea, there is sparse data concerning FMD in healthy people. In the study by Park et al., the average of FMD in 25 healthy people, a mean aged 54 years, was 14.3% [17]. The study also showed that FMD was 18.0% in 13 young healthy people, a mean age of 26 years. In the study by Bae et al., it showed FMD in 10 young healthy people, a mean age of 26 years, was 13.0% [18]. These studies showed the differences in FMD measurement compared with the results of our study, although brachial artery diameters were similar in our study (3.6 and 3.58 vs 3.61 mm). Since the experimental group was too small in these studies including our own study, it probably led to have an increase in bias and difference of FMD. Moreover, the automatic measurement is more reproducible and less subjective [19]. In the two studies mentioned above, FMD was analyzed by calipers without a system for the automatic evaluation. But we analyzed FMD with automatic measurement system. Therefore, we should consider a probable bias from manual measurement in the two studies mentioned above. In the study by Hwang et al., it showed FMD was 8.98% in 74 young healthy volunteers, with a mean age 22 years [16]. There was no any significant difference compared with the 8.45% from our study. In the study by Vlachopoulos et al., it showed FMD was about 6.0% in healthy people, with a mean age 32 years [11]. However, only 12 subjects participated in this study. Therefore, a large scale study should be performed to achieve precise results.

In our present study, the results show the reproducibility of the FMD measurement regardless of the time of day, and it is not necessary to standardize the time of FMD measurement. It is widely known that a principle mediator of FMD is endothelium-derived nitric oxide (NO) [20,21]. In addition, there is a close interaction between the sympathetic nervous system and NO [22]. Diurnal changes in sympathetic activity are generally held responsible for diurnal changes in NO availability. However, in our study, sympathetic activity such as systolic blood pressure, diastolic blood pressure, and heart rate was similar in the morning and afternoon. Thus, we assume that it may lead to insignificant diurnal variation between FMD in the morning and afternoon.

Moreover, the concept that the observed diurnal pattern of FMD reflected a diurnal variation in NO production was not supported in some studies. In the study by Järvisalo et al., the plasma levels of NO degradation products showed significant variation with the highest levels in the morning and lowest in the afternoon but were not significantly associated with FMD [23]. In a previous study in healthy pre-menopausal women, the urinary excretion or plasma levels of nitrate did not display diurnal variation or associate with FMD [15]. Thus, it will be necessary to continue the research in a large-scale and well-controlled study.

To standardize the assessment of FMD, most study groups have performed the measurements in the morning on fasting patients. However, it leads to a limit of the wide-scale use of this methodology in routine clinical practices. Previous studies showed that postprandial changed in endothelial function are controversial [11,12,24]. Our study was well controlled for diet, as a result, triglyceride and glucose levels were similar in the morning and afternoon. Therefore, it is reasonable to assume that diet is a major factor effect on FMD compared with the time of measurement on FMD.

Our study has some limitations. Firstly, laboratory findings such as total cholesterol, triglyceride, HDL, LDL, and glucose were only analyzed by capillary blood. Even if samplings of capillary blood showed no difference in the two recordings, it did not represent the serum levels. To overcome this limitation, we provided a sufficient fasting period to all subjects. Secondly, we did not consider intra-observer variability of FMD. However, we did check FMD by automatic measurement. We believe that it leads to more reproducible and less subjective results.

## Conclusion

Measurement of FMD is 8.45% in healthy Koreans, and our results show there were no significant differences between FMD in the morning and afternoon. It will be helpful to evaluate endothelial function in routine clinical practice.

## Abbreviations

2D: two-dimensional
FMD: flow-mediated dilatation
HDL: high-density lipoprotein
LDL: low-density lipoprotein
NO: nitric oxide
SD: standard deviation
